# The cysteine protease ATG4B of *Trichinella spiralis* promotes larval invasion into the intestine of the host

**DOI:** 10.1186/s13567-020-00791-z

**Published:** 2020-05-24

**Authors:** Yalan Li, Baiyan Wang, Yaxin Zhu, Zhihua Tian, Zhuo Yang, Jiaqi Duan, Zhongquan Wang

**Affiliations:** 1 Department of Pathogen Biology, School of Basic Medical Sciences, Zhengzhou University, Zhengzhou, China; 2 Scientific Research Experimental Center, School of Basic Medical Sciences, Henan University of Chinese Medicine, Zhengzhou, China

## Abstract

The cysteine proteases of parasites are vital contributors that induce parasite migration to and invasion of host tissue. In this study, we analysed the cysteine protease ATG4B of *Trichinella spiralis* (TsATG4B) isolated from the soluble proteins of *Trichinella spiralis* (*T. spiralis*) adult worms to ascertain its biochemical properties and functions during invasion into the intestine of the host. The 43 kDa recombinant cysteine protease ATG4B protein (rTsATG4B) consists of a conserved peptidase_C54 domain and was expressed in *Escherichia coli*. Gelatine zymography showed that rTsATG4B could hydrolyse gelatine and that the hydrolytic activity was prevented by the cysteine protease inhibitor E-64 (pH 5.2). Immunofluorescence assays showed that TsATG4B is expressed at different stages and is localized at the cuticles and stichosomes of worms. Far-Western blotting and confocal microscopy revealed that rTsATG4B interacts with intestinal epithelial cells (IECs) and that it was subcellularly localized to the membrane and cytoplasm in IECs. Real‑time quantitative PCR (qPCR) results indicated that the transcription level of the TsATG4B gene was the higher in 6-day-old adult worms (6 days AW) than in any other stage. An in vitro larval invasion assay verified that rTsATG4B promoted larval invasion and that invasion was inhibited when rTsATG4B was pre-incubated with E-64, whereas anti-rTsATG4B serum inhibited larval invasion in a dose-dependent manner. Collectively, these results suggested that the enzymatic activity of TsATG4B significantly influences the hydrolysis process, which is necessary for larval invasion of the host intestinal epithelium.

## Introduction

Trichinellosis is a global food-borne parasitic zoonosis that remains an emerging disease that has been threatening public health and affecting economic growth [1–3]. Infection is initiated when uncooked or raw animal meat that has been contaminated with *Trichinella spiralis* (*T. spiralis*) is consumed [4, 5]. After exposure to gastric juice in the gastrointestinal environment, *T. spiralis* muscle larvae (ML) are released from the capsules in the stomach. The worms grow by relying on intestinal contents, and they develop into intestinal infective larvae (IIL) in the intestine. Subsequently, IIL invade the epithelium of the small intestine, where they undergo 4 moults before developing into adult worms (AW), and they then mate and produce newborn larvae (NBL). NBL travel through the blood and lymph from the intestine to striated muscle, where they finally develop into L1 stage larvae in muscle cells [6, 7].

At the intestinal infection stage, the helminths establish an intramural niche with numerous epithelial cells and localize at the crypt-villus junction. When the nematodes can migrate in a sinusoidal pattern through the epithelium, they invade and inhabit the cytoplasm of new cells, leaving trails of dead cells behind [8]. *T. spiralis* larvae have no visible tools to promote their invasion, such as oral spikes, and the mechanisms by which *T. spiralis* larvae recognize, migrate to and invade the intestinal epithelium are not clear [9]. However, it has been reported that the mechanisms of larval invasion into the intestinal epithelium are not simply related to mechanical penetration but are related to the surface and oral secretory proteins of the worms [10, 11]. To successfully breach the barrier of the intestinal epithelium, parasites must effectively degrade various host proteins but minimize tissue damage to reduce innate immune responses in order to swiftly and successfully infect the host [12]. Many parasitic helminths can utilize an array of host proteins, especially haemoglobin, as the principal source of amino acids. During this process, cysteine proteases are the key proteases of the helminths that degrade haemoglobin into amino acids [13]. Timms and Bueding [14] first described the proteases in *schistosome* extracts with an acidic optimum pH. Currently, it is known that many proteases that play important roles in the degradation of haemoglobin into free amino acids, including cathepsin D (an aspartic protease of clan AA) and cathepsins B1, C, L1/F, L2, and L3 (papain-like cysteine proteases of clan CA, family C1) are secreted into the *schistosome* intestinal tract; thus, these proteases are highlighted as important drug targets [15, 16].

*T. spiralis* expresses different kinds of immunodominant antigens during all developmental stages [17]. These proteins have been verified to play critical roles in larval invasion and host immune system modulation, as well as in facilitating the establishment of parasitism and *T. spiralis* survival [18–22]. Moreover, research has shown that cysteine proteases play crucial roles in the invasion and migration of helminths throughout the host tissue [23, 24]. Cysteine proteases from parasitic organisms can effectively degrade host tissue to promote the penetration and migration of helminths at various stages of parasite development; thus, they are vital contributors to these processes [12]. The cysteine protease ATG4B of *T. spiralis* belongs to the C54 peptidase family (Aut2 peptidase family, clan CA) [25]. TsATG4B protein, which is recognized in early *T. spiralis*-infected sera, and has also been suggested to be an immunodiagnostic antigen and a promising vaccine target, was isolated from the soluble protein of *T. spiralis* AW [23, 26]. Therefore, the purpose of this study was to ascertain the biochemical characteristics and functions of TsATG4B during the process of invasion of the host intestine.

## Materials and methods

### Experimental animal housing conditions and parasite maintenance

Experimental animals (BALB/c mice and Kunming mice) were purchased from the Experimental Animal Center of Henan Province, China. All experimental procedures were reviewed and approved by the Life Science Ethics Committee of Henan Province (ethics approval number DWLL 201903062). The *T. spiralis* ISS 534 strain [27, 28] was initially obtained from domestic pigs in Henan Province, China, and serialized in Kunming female mice. Muscle larvae were obtained from the muscle of infected Kunming mice by artificial digestion [29]. AW were collected from the intestines of mice 3 and 6 days (d) after experimental infection. The 6 days AW were cultured in Roswell Park Memorial Institute (RPMI)-1640 medium for 24 h, and NBL were harvested from the culture. Excretory-secretory (ES) products and soluble proteins of *T. spiralis* were prepared according to methods described in our previous study [30].

### Cell culture and cell lysate protein preparation

Normal mouse intestinal epithelial cells (IECs) were previously isolated from BALB/c mouse intestines and maintained for the in vitro larval invasion assay [29]. Investigations revealed that the IECs were susceptible to larval invasion of *T. spiralis*, in contrast to C2C12 cells from mouse striated muscle myoblasts, which were resistant to larval invasion. IECs were used in the in vitro larval invasion assay, and C2C12 cells were used as a negative control. IECs and C2C12 cells were cultured in 25 cm^2^ cell culture flasks (Corning, NY, USA) in Dulbecco’s modified Eagle’s medium (DMEM) (Solarbio, Beijing, China) supplemented with 5% foetal bovine serum (FBS) (Solarbio, China) and incubated in 5% CO_2_ at 37 °C. To maintain the cell cultures, the medium was renewed every 2 or 3 days, and cell monolayers were digested with 0.25% trypsin (Solarbio, China). Preparation of proteins from IEC and C2C12 lysates was performed as described above [31].

### Bioinformatic analysis of TsATG4B

According to the information provided by the National Center for Biotechnology Information (NCBI), the access number of the *TsATG4B* gene is XM_003371464.1. The access number for the TsATG4B protein is XP_003371512.1. Conserved domain analysis of TsATG4B was performed with the Conserved Domain database of NCBI. The open reading frames (ORFs) were identified in ORF Finder. The basic theoretical characterization of proteins, the molecular weight and the isoelectric point were analysed by bioinformatics software and web servers. SignalP4.1 Server was used to predict the signal peptide. The transmembrane domain was predicted by TMHMM Server v. 2.0. The TsATG4B protein domains were analysed with EMBL-EBI software. The B cell epitope was analysed by an online server. The phylogenetic tree of TsATG4B and homologous sequences was constructed by MEGA 7.0, and the phylogeny was constructed with the maximum parsimony method.

### Cloning, expression, purification and refolding of recombinant TsATG4B

To analyse the biochemical properties of TsATG4B, the full length TsATG4B gene (XM_003371464), which is 1245 bp long, was initially synthesized, and the recombinant protein was expressed in *Escherichia coli*. PCR was performed to amplify the region comprising amino acid residues 18-414 with the following primers: 5′-ACCATCACCATCACGGATCCCGATTGGAACTGCTCGACGA-3′ (the BamHI restriction site is underlined) and 5′-AAGCTCAGCTAATTAAGCTTTCATTCAAAACCAACTTCAGATAT-3′ (the HindIII restriction site is underlined). The thermal cycling procedure was as follows: predenaturation at 94 °C for 3 min; 30 cycles at 94 °C for 30 s, 60 °C for 30 s, and 72 °C for 90 s; and final extension at 72 °C for 5 min. The PCR product was subcloned into a pQE-80L expression vector containing a His tag at the C-terminus (Novagen, La Jolla, USA) following the protocol of the ClonExpress II single-pass clone kit (Vazyme, Nanjing, China), and the recombinant plasmid pQE-80L/TsATG4B was then transformed into *Escherichia coli* BL21 (Novagen, La Jolla, USA) via induction with 0.5 mM isopropyl-1-thio-β-d-galactopyranoside (IPTG) (Sangon Biotech Co., Shanghai, China) for 4 h at 37 °C for protein expression.

To purify rTsATG4B, cells were harvested and lysed by sonication in a 200 W sonicator for 4 s and were then placed in ice water for a total of 200 cycles at 2 s per cycle. Then, rTsATG4B was centrifuged at 12 000 *g* and 4 °C for 30 min. rTsATG4B was mostly aggregated in inclusion bodies and was analysed by SDS-PAGE with a 10% acrylamide separating gel [13]. Then, the supernatant was discarded, and the inclusion body pellets were collected and resuspended in 20 mL of ice-cold inclusion body buffer solution containing 50 mM Tris–HCl, 8 M urea, 1 mM ethylenediaminetetraacetic acid (EDTA), and 100 mM NaCl (pH 8.0) to dissolve inclusion bodies [33]. The denatured protein was purified by metal affinity chromatography with Ni–NTA-Sefinose resin (Sangon Biotech Co., China) under denaturing conditions, according to the manufacturer’s instructions [33]. SDS-PAGE separates proteins according to their molecular weight based on their differential rates of migration through a sieving matrix (gel) under the influence of an applied electrical field. Therefore, the expression and purification of rTsATG4B protein were confirmed by SDS-PAGE, and a single band with a molecular weight of 43 kDa was observed on the SDS-PAGE gel (Figure 2A).

Via the “one-step-denaturing and refolding method” [32], purified rTsATG4B dissolved in inclusion body solubilization buffer was first placed in a dialysis bag (8000–14 000 kDa, Solarbio, China), and the dialysis process was carried out with PBS medium for 4 h. The PBS dialysis medium was kept cold to generate a clean and completely folded protein. Subsequently, the refolded protein was concentrated by ultrafiltration at 5000 *g* for 30 min (Millipore, Billerica, USA). After a second analysis by SDS-PAGE, the molecular weight of the heated refolded rTsATG4B was 43 kDa, which corresponded to the theoretical molecular weight calculated for the amino acid sequence.

### Production of infection sera and the anti-TsATG4B polyclonal antibody and identification of TsATG4B by Western blot analysis

Infection serum was obtained from BALB/c mice that were orally administered 300 *T. spiralis* for 3, 6, 10, 14, 17, 20, 23, 26, 30, 36 and 42 days. Purified TsATG4B protein (20 μg) was injected subcutaneously into one BALB/c mouse as a formulation with complete Freund’s adjuvant (FA, Sigma, St. Louis, USA) according to the instructions. Two more injections of the same dose of rTsATG4B protein mixed with incomplete FA (Sigma, USA) were administered to mice over a 2-week period. Fourteen days after the last immunization, immune sera were collected, and the antibody titres were determined by ELISA [33].

Soluble proteins of ML, IIL, 3 days AW, 6 d AW, and NBL and ES proteins of ML were separated on 10% polyacrylamide SDS-PAGE gels. After electrophoresis, the proteins were transferred to nitrocellulose membranes (Millipore, USA). Membranes were blocked with 5% nonfat dry milk, incubated with TsATG4B immune serum, *T. spiralis*-infected mouse serum or pre-immune mouse serum at 1:100 dilutions overnight at 4 °C; washed with PBST three times for 10 min each, incubated with a horseradish peroxidase (HRP)-conjugated goat anti-mouse antibody (Sangon Biotech Co., China) at a 1:5000 dilution at 37 °C for 1 h, and washed 3 more times with PBST for 10 min each. Detection of protein bands was performed with 3,3′-diaminobenzidine tetrahydrochloride (DAB, Sigma, USA) in the dark. Images were acquired with an Image Scanner III (GE Healthcare, Chicago, USA) [31].

### Detection of refolded rTsATG4B activity by zymography

For gelatine zymography, 10% SDS–PAGE gels copolymerized with 1 mg/mL gelatine (Sigma) were used. To test the effect of the E-64 inhibitor, refolded rTsATG4B protein samples were incubated with E-64 (10 mM) at a ratio of 1:1 (v/v) at 37 °C for 30 min. Then, 25 μL of each supernatant sample was loaded into each well under non-reducing (native) conditions, and samples were separated at 120 V for 2 h. After electrophoresis, gels were transferred to clean containers and washed two times for 60 min each with 100 mL of 2.5% (v/v) Triton X-100 by gently shaking on a rotary stirrer at room temperature, and then the washes were repeated with distilled water three times. After incubating in acetate buffer (pH 5.2) with 1 mM dithiothreitol for 36 h at 37 °C, gels were washed three times with distilled water to remove the activation buffer. Next, gels were stained with Coomassie blue solution (Sangon Biotech Co., China) under constant stirring for 2 h. Finally, gels were placed in a bleaching solution with 7% acetic acid and 5% methanol for 1 h to identify the bands with proteolytic activity [34–36]. The same samples were separated by 10% SDS-PAGE under non-reducing (native) conditions, and proteins were then transferred to nitrocellulose membranes. Western blot detection was performed as mentioned above. Images were acquired with an Image Scanner III (GE Healthcare, USA).

### Real-time quantitative PCR (qPCR) analysis of *TsATG4B* gene transcription

Total RNA from ML, IIL, 3 days AW, 6 days AW and NBL was extracted by TRIZOL reagent (Invitrogen™, Carlsbad, USA) and reverse transcribed with a Prime Script RT Reagent Kit with gDNA Eraser (Takara, Tokyo, Japan). The *T. spiralis 18S rRNA* gene (GenBank accession number U60231) was used as the internal control with the primers 5′-CAACCTTCGATGGTAGCCTATGCG-3′ and 5′-CCTGCTGCCTTCCTTGGATGTG-3′, and the predicted length was 117 bp [33]. The primers 5′-TCCCCATTATAGTCAACCTGCT-3′ and 5′-TGGATATTTACAATGAAAACTGTGAAG-3′ were designed to be specific for the *TsATG4B* gene, with the predicted length of the product being 76 bp. qPCR was performed in a 20 µL reaction mixture containing SYBR Premix (Takara, Japan), cDNA, primers, and ROX Reference Dye II in an ABI 7300 real-time PCR system (Applied Biosystems, Foster City, USA). The different transcription levels were calculated with the formula 2^−ΔΔCt^ [37]. The experiment was repeated three times (*n* = 3).

### Expression and immunolocalization of TsATG4B at various stages by an immunofluorescence test

An immunofluorescence test (IFT) was used to determine the localization of the native TsATG4B protein at various *T. spiralis* developmental stages, and paraffin sections were also tested. Live *T. spiralis* worms at different developmental stages (ML, IIL, AW and NBL) were fixed with paraformaldehyde for 30 min after being washed three times, fixed with cool acetone for 20 min, and subsequently washed three more times. The worms and paraffin sections were blocked with 5% normal goat serum and incubated in a humidified chamber at 37 °C for 1 h. The goat serum was then discarded, and the slides with worms were incubated with pre-immune mouse serum diluted 20-fold (negative control), *T. spiralis* infection serum (positive control) and the anti-TsATG4B polyclonal antibody at 37 °C for 1 h. After three washes with PBS, the slides and worms were incubated with FITC-labelled goat anti-mouse IgG (Sigma, USA) at a 1:100 dilution at 37 °C for 45 min. After being washed, the slides and worms were observed under a fluorescence microscope (Olympus, Tokyo, Japan), and images were acquired.

### Immunofluorescence assay and Far-Western blot

For the IEC and C2C12 cell immunofluorescence assays, IECs and C2C12 cells were grown in DMEM containing 10% FBS (Solarbio, China). IEC and C2C12 cells were seeded after digestion with trypsin (0.25%, Solarbio, China) on glass coverslips coated with poly-l-lysine (Solarbio, China). Cells were grown in a 6-well cell culture plate for 36 h to a confluence of approximately 90%. The culture medium was discarded, and the glass coverslips were washed three times with PBS, fixed in acetone for 10 min, blocked with 5% goat serum at 37 °C for 2 h, and finally incubated with 20 μg/mL TsATG4B (at 37 °C for 2 h, with PBS as the negative control). After three washes with PBS, cells were probed with the first and second antibodies, following the previously mentioned IFT protocols. Images were acquired with a laser scanning microscope (Olympus FV1200, Olympus, Tokyo, Japan).

The protein–protein interaction between TsATG4B and IECs was analysed by Far-Western blotting. After the proteins in the samples of IEC and C2C12 cell lysates were separated by SDS-PAGE with 10% polyacrylamide gels, the proteins were transferred to NC membranes. The NC membranes were incubated with 20 μg/mL rTsATG4B for 1 h at 37 °C and then analysed by Western blotting. After the membranes were washed, the protein bands were stained with DAB (Sigma, USA) in the dark, and the reaction was terminated with distilled water. Images were acquired with an Image Scanner III (GE Healthcare, USA).

### In vitro invasion assay

To evaluate the effects of rTsATG4B and anti-rTsATG4B serum on larval invasion of the intestinal epithelium, a *T. spiralis* invasion test was performed in vitro. IECs were cultured to full confluence in 6-well plates. ML were stimulated to develop into IIL by treatment with 5% porcine bile at 37 °C for 2 h [8]. Two groups of TsATG4B with different concentrations ranging from 2.5 to 15 μg/mL were prepared, and one group was pre-incubated with E-64 (10 μM, 1:1) at 37 °C for 30 min. Subsequently, 1:50 to 1:1600 dilutions of serum (anti-TsATG4B serum, *T. spiralis*-infected mouse serum and pre-immune mouse serum) were prepared. Then, the samples of each treatment group were added to 1.75% agarose semi-solid medium containing 100 IIL and gently mixed at 38 °C to 40 °C. The mixed medium was overlaid on the monolayer of IECs in each well and formed a semi-solid medium. After incubation in 5% CO_2_ at 37 °C for 2 h, larval invasion of IECs in each treatment group was observed. Larvae that invaded or migrated in the IEC monolayer showed trails, leaving dead cells behind and destroying the IEC monolayer. Larvae suspended in the medium or on the cell monolayer surface, which did not stretch or damage the cell monolayer, were defined as uninvaded larvae. Experiments were conducted in triplicate [31, 35].

### Statistical analysis

The data were analysed statistically with IBM SPSS Statistics 25.0 for Windows (IBM Corporation, NY, USA). The relative expression data of the *TsATG4B* gene in ML, IIL, 3 days AW, 6 days AW and NBL are displayed as the mean ± SD values, and one-way ANOVA was used to compare the differences among the groups. The different percentages of invaded larvae in the IEC monolayers with different concentrations (0–15 μg/mL) of TsATG4B and different serum dilutions were analysed using a Chi square test. Statistical significance was defined as *P* < 0.05.

## Results

### Bioinformatic analysis of TsATG4B and phylogenetic analysis of TsATG4B

**Figure 1:**
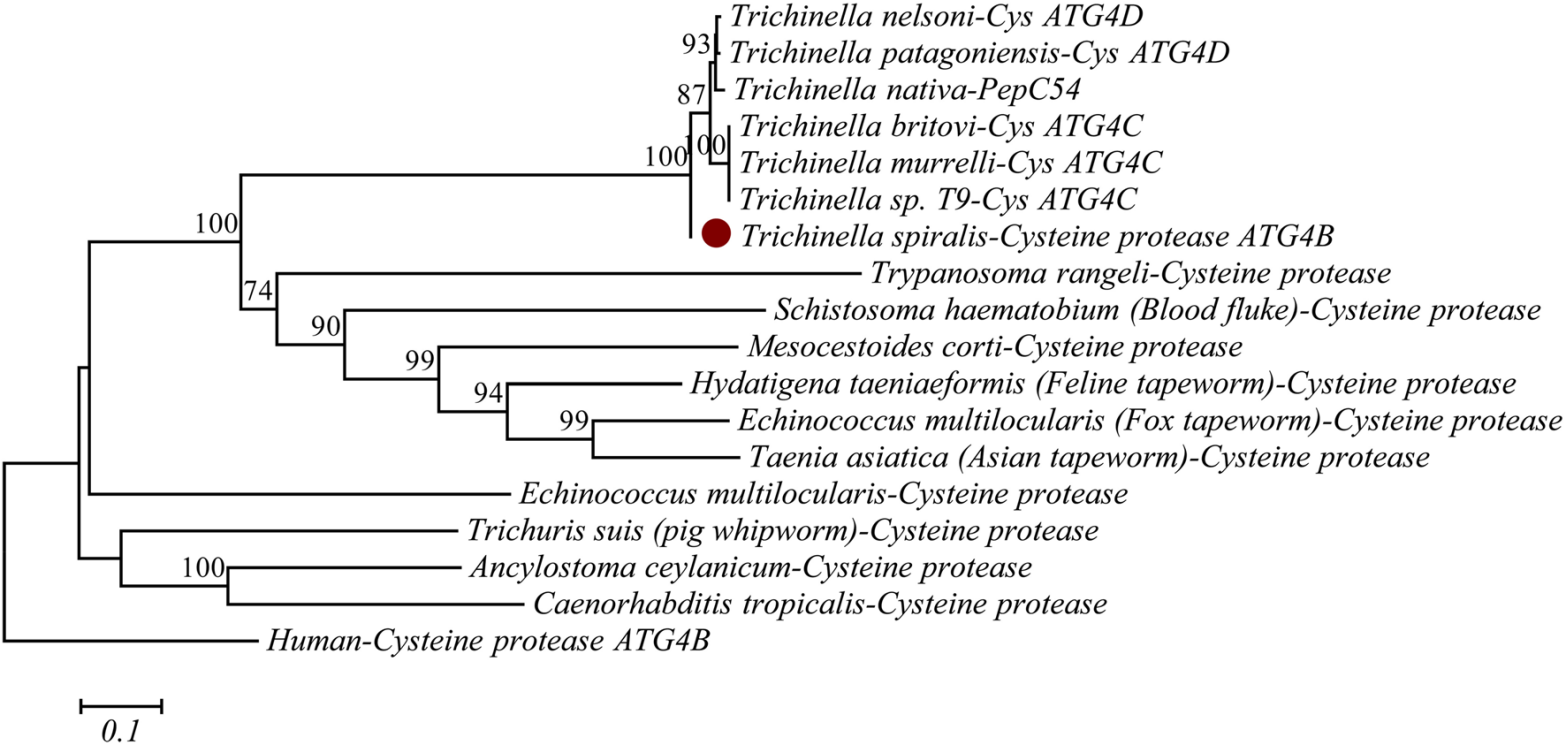
**Analysis of the phylogenetic tree of TsATG4B. **Phylogenetic relationship of the peptidase_C54 domain of nematodes and humans using the maximum parsimony method and drawn by MEGA. Bootstrap values of less than 70 are hidden in the branches. The sequence denoted by a solid circle is that of the TsATG4B protein expressed in this study.

The complete CDS of *TsATG4B* (XM_003371464.1) contains an open reading frame of 1245 bp encoding a protein of 414 aa that contains a conserved peptidase_C54 domain (aa 79–404) and has a calculated molecular weight of 46.94 kDa and an isoelectric point of 5.79. Signal P 4.1 Server prediction showed that there was no signal peptide and that the protein was a non-secretory protein. It was predicted that aa 33–43, aa 55–62, aa 176–182, aa 236–244, aa 246–255, aa 261–268, aa 271–277, and aa 286–293 were B-cell epitopes. Phylogenetic analysis of the peptidase_C54 domain of different kinds of organisms favoured the monophyletic group of six species of *Trichinella* (*Trichinella nelson*, *Trichinella patagoniensis*, *Trichinella native*, *Trichinella murrelli*, *Trichinella britovi*, and *Trichinella sp. T9*) (Figure 1).

### Purification and gelatinolytic activity of rTsATG4B

**Figure 2:**
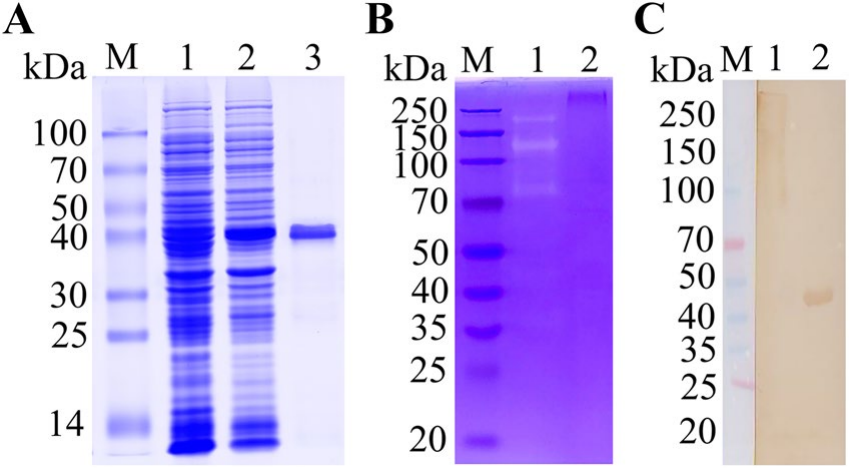
**Expression, purification and gelatinolytic activity of rTsATG4B. A** Expression and purification of rTsATG4B. Lane M: standard marker; lanes 1/2, lysate of BL21 bacteria harbouring pQE-80L/TsATG4B without/with IPTG induction; lane 3: purified recombinant TsATG4B; **B** Proteolytic activity of refolded TsATG4B as detected by gelatine zymography. Lane M: protein molecular weight marker; lane 1: transparent strips due to the proteolytic activity of refolded TsATG4B in acrylamide-gelatine gels; lane 2: refolded rTsATG4B pre-incubated with the protease inhibitor E-64; **C** Identification of refolded rTsATG4B by Western blotting. Lane M: protein molecular weight marker; lane 1: refolded rTsATG4B recognized by anti-TsATG4B serum (1:100 dilution); lane 2: denatured rTsATG4B recognized by anti-TsATG4B serum (1:100 dilution).

To analyse the biochemical and immunogenic properties of TsATG4B, the amplified fragment (1194 bp, containing the conserved peptidase_C54 domain) was cloned into the pQE-80L expression vector with a seamless clone kit. The TsATG4B protein fused with a His-tag was expressed in *E. coli* cells and purified with Ni-affinity chromatography. After analysis by SDS-PAGE, a single band was observed on the SDS-PAGE gel at a molecular weight of approximately 43 kDa, consistent with the calculated molecular weight (Figure 2A, lane 3). In the 1-D gelatine zymogram of purified rTsATG4B, the clear band revealed gelatinolytic activity (Figure 2B, lane 1) and was eliminated by treatment with E-64 (Figure 2B, lane 2), a cysteine protease inhibitor. Furthermore, the band of refolded rTsATG4B was at the same position and was recognized by anti-TsATG4B serum (1:100 dilution), as indicated by Western blot analysis (Figure 2C). This result showed that the hydrolysed bands of gelatine were generated due to the hydrolytic activity of rTsATG4B.

### Identification of TsATG4B

**Figure 3:**
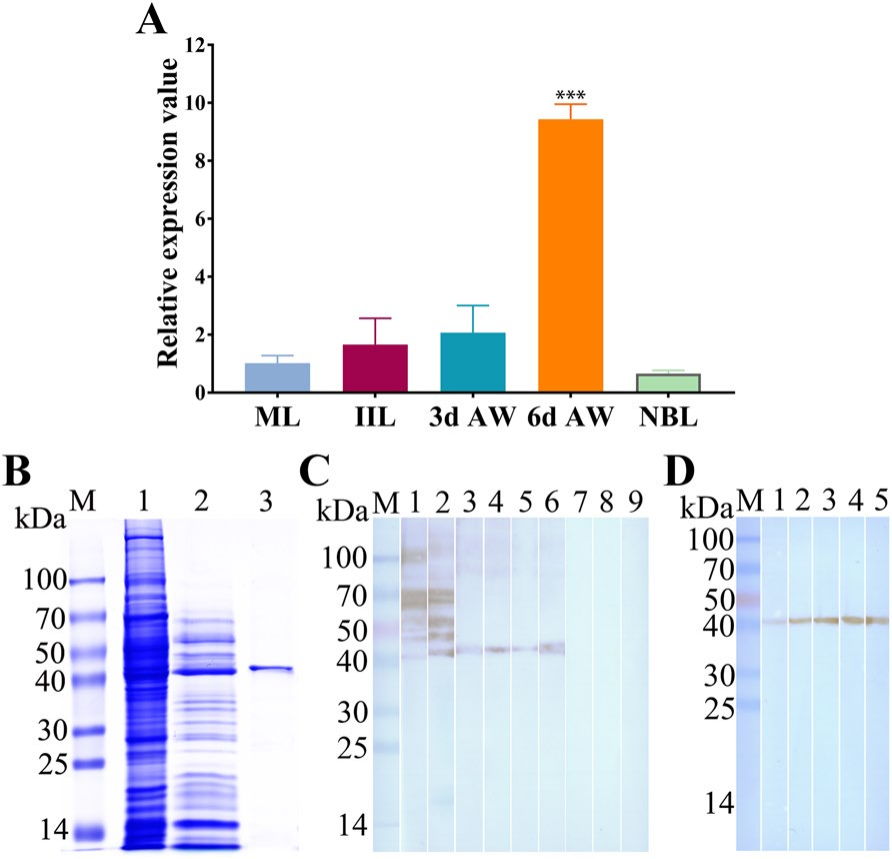
**Identification and expression of TsATG4B. A** qPCR analysis of *TsATG4B* gene transcription levels at various stages of *T. spiralis* development. 18S rRNA was used as the housekeeping gene control. ***P < 0.001, compared to the level in ML. **B** Results of TsATG4B analysis by SDS-PAGE. Lane M: protein molecular weight marker; lane 1: somatic proteins of *T. spiralis* ML; lane 2: ES proteins of ML; lane 3: purified rTsATG4B protein product; **C** Analysis of rTsATG4B antigenicity by Western blotting. Lanes 1, 4, and 7: soluble proteins from ML; lanes 2, 5, and 8: ES proteins of ML; lanes 3, 6, and 9: purified rTsATG4B. Proteins were probed with infection serum (1:100 dilution, lanes 1, 2, and 3), anti-TsATG4B serum (1:100 dilution, lanes 4, 5, and 6) and normal mouse serum (1:100 dilution, lanes 7, 8, and 9). **D** Native TsATG4B in soluble proteins of different developmental stages of *T. spiralis* (lane 1: NBL; lane 2: ML; lane 3: IIL; lane 4: 3 days AW; lane 5: 6 days AW) was recognized by anti-TsATG4B serum.

The qPCR results indicated that the *TsATG4B* gene was transcribed during different stages (NBL, ML, IIL, 3 days AW and 6 days AW) (Figure 3A). The transcription level of the *TsATG4B* gene in 6 days AW was significantly higher than that in ML, IIL, 3 days AW and NBL (F (4) = 98.144, *P *< 0.01). The amplicons of the *TsATG4B* gene and *T. spiralis 18S rRNA* gene were displayed as 76 bp and 117 bp, respectively, by 5% agarose gel electrophoresis (Additional file 1).

The Western blot results showed that the rTsATG4B protein was recognized by anti-rTsATG4B serum and serum from mice infected for 20–30 days. The anti-TsATG4B polyclonal antibody recognized the native TsATG4B protein among somatic proteins from NBL, ML, IIL, 3 days AW and 6 days AW and ES products from ML, as indicated by Western blot analysis, illustrating that the TsATG4B protein was a constituent of these proteins (Figures 3C, D).

**Figure 4:**
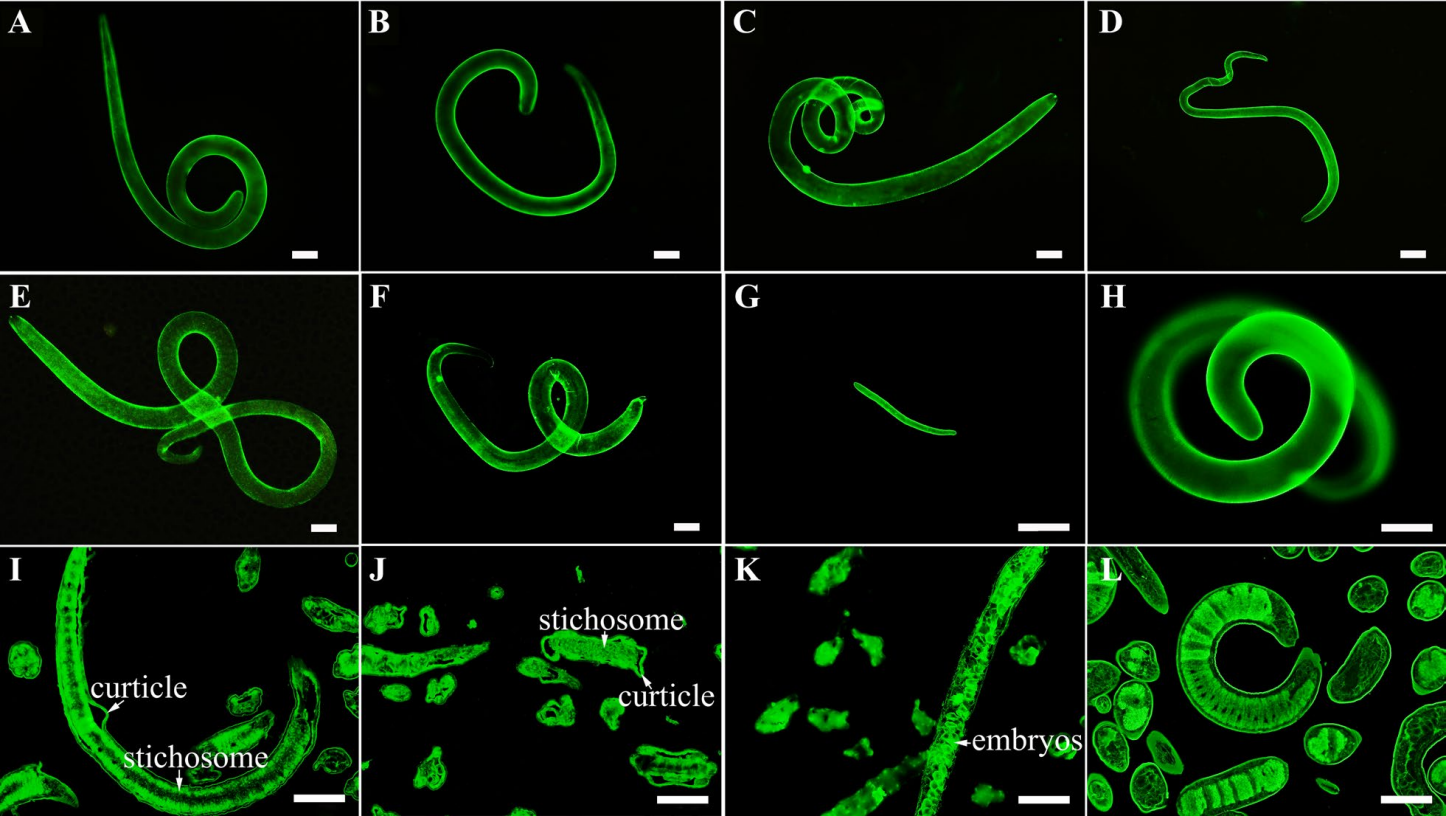
**Identification and expression of TsATG4B in the various developmental stages by immunolocalization. A**–**H** Intact worms were recognized by anti-rTsATG4B mouse serum, as shown by IFA. Bright immunostaining was displayed on the surface of ML **A**, IIL **B**, 3 days female AW **C**, 3 days male AW **D**, 6 days female AW **E**, 6 days male AW **F**, and NBL **G**. ML **H** were probed with infection serum as a positive control. **i**–**l** Paraffin sections of *Trichinella spiralis* were recognized by anti-rTsATG4B mouse serum, as shown by IFA. Bright immunostaining was displayed on the cuticles and stichosomes of ML **I** and IIL **J** and in the embryos of 3 days female AW **K**. Paraffin sections of *Trichinella spiralis* ML **L** probed with infection serum were used as a positive control. Scale bars: 50 μm.

The results of IFT revealed intense immunostaining in the cuticles of intact worms and that TsATG4B was expressed at different *T. spiralis* stages (Figure 4). Bright immunostaining was observed on the cuticles, surfaces and stichosomes of the paraffin sections of the worms incubated with immune serum and infection serum (Figure 4), but no immunostaining was observed in the worms incubated with pre-immune serum or PBS (Additional file 2).

### Interaction of TsATG4B with IECs by immunofluorescence assay and Far-Western blot analysis

**Figure 5:**
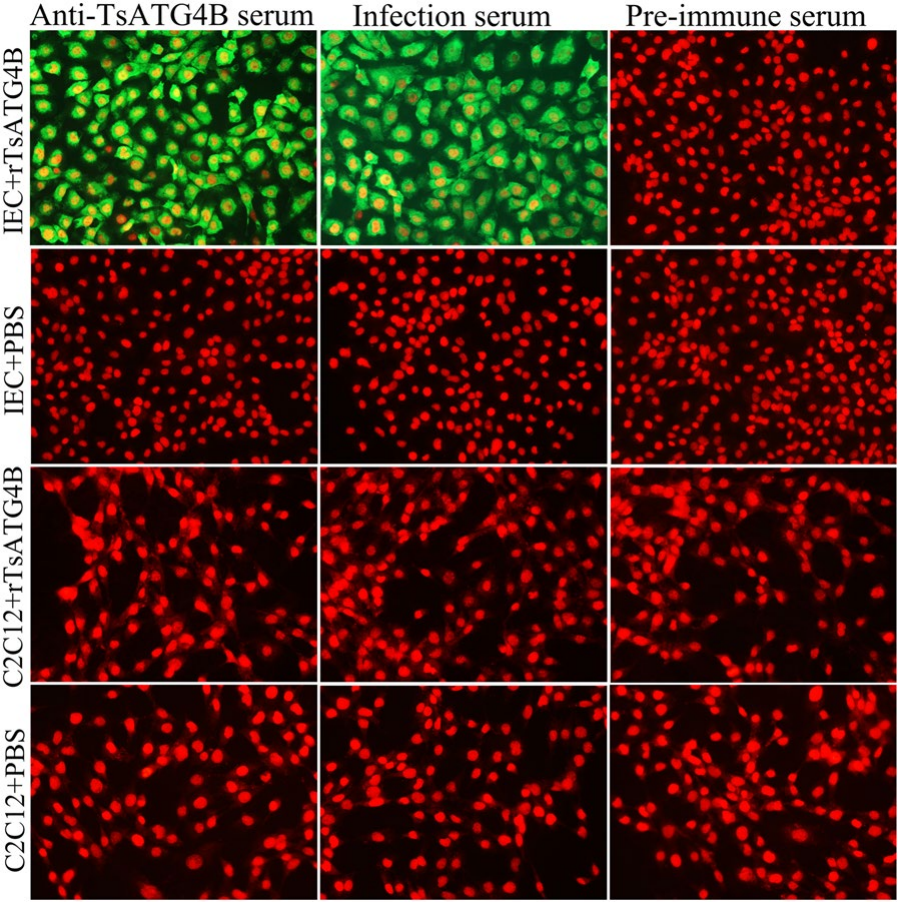
**Immunofluorescence assay of rTsATG4B binding to IECs (200×). **IECs and C2C12 cells were pre-incubated with rTsATG4B at 37 °C for 2 h. Then, they were probed with different antibodies (anti-TsATG4B polyclonal antibody, *T. spiralis*-infected mouse serum and pre-immune mouse serum) and the FITC-conjugated goat anti-mouse IgG secondary antibody and were then counterstained with propidium iodide (PI; the fluorescent dye PI is a nuclear staining reagent that can stain the nucleus through the disrupted cell membrane and emits red fluorescence after embedding in double-stranded DNA).

**Figure 6:**
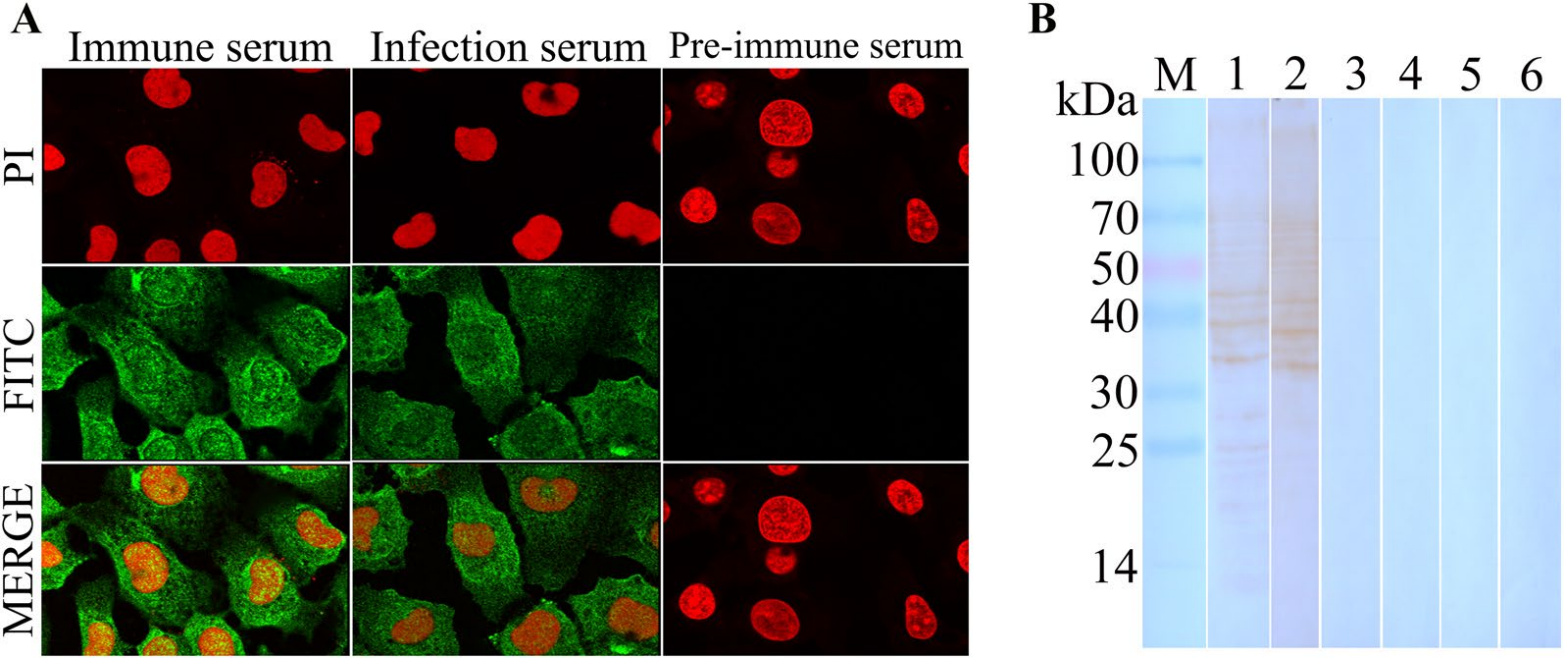
**Interaction of TsATG4B with IECs by confocal microscopy and Far-Western blot analysis. A** The subcellular localization of rTsATG4B bound to IECs was observed via confocal microscopy (1000×). IECs were pre-incubated with rTsATG4B for 2 h at 37 °C, and then the same procedure used in the immunofluorescence assay was performed. Images were acquired and analysed with an Olympus FV1200 laser scanning microscope. **B** Analysis of rTsATG4B binding to IEC proteins by Far-Western blotting. After the IEC lysates (lanes 1–3) and C2C12 lysates (lanes 4–6) were transferred to NC membranes, the NC membranes were incubated with rTsATG4B for 2 h at 37 °C. Then, the membranes were individually probed with anti-TsATG4B serum (lanes 1, 4), infection serum (lanes 2, 5) and pre-immune serum (lanes 3, 6). Binding between rTsATG4B and IEC lysates was detected with anti-TsATG4B serum (lane 1) and infection serum (lane 2).

Fluorescence staining was observed on the surface of IECs incubated with rTsATG4B and probed with anti-rTsATG4B serum and infection serum but was not detected on IECs probed with normal serum. Fluorescence staining was not observed on the surface of C2C12 cells incubated with rTsATG4B (Figure 5). Confocal microscopy revealed that rTsATG4B could localize to the membrane and cytoplasm of IECs (Figure 6A). Analysis of soluble proteins from IECs and C2C12 cells by SDS-PAGE showed approximately 20–30 bands from 14 to 100 kDa (Additional file 3). The Far-Western blot results of IEC lysates pre-incubated with rTsATG4B showed that there were approximately 10 bands (20–70 kDa) on the membranes probed with anti-rTsATG4B serum and infection serum but not with normal serum. C2C12 cell lysates pre-incubated with rTsATG4B were not recognized by anti-TsATG4B serum, infection serum or normal serum (Figure 6B).

### Promotive or inhibitory effects of TsATG4B or anti-rTsATG4B serum on larval invasion of IEC monolayers in vitro

**Figure 7:**
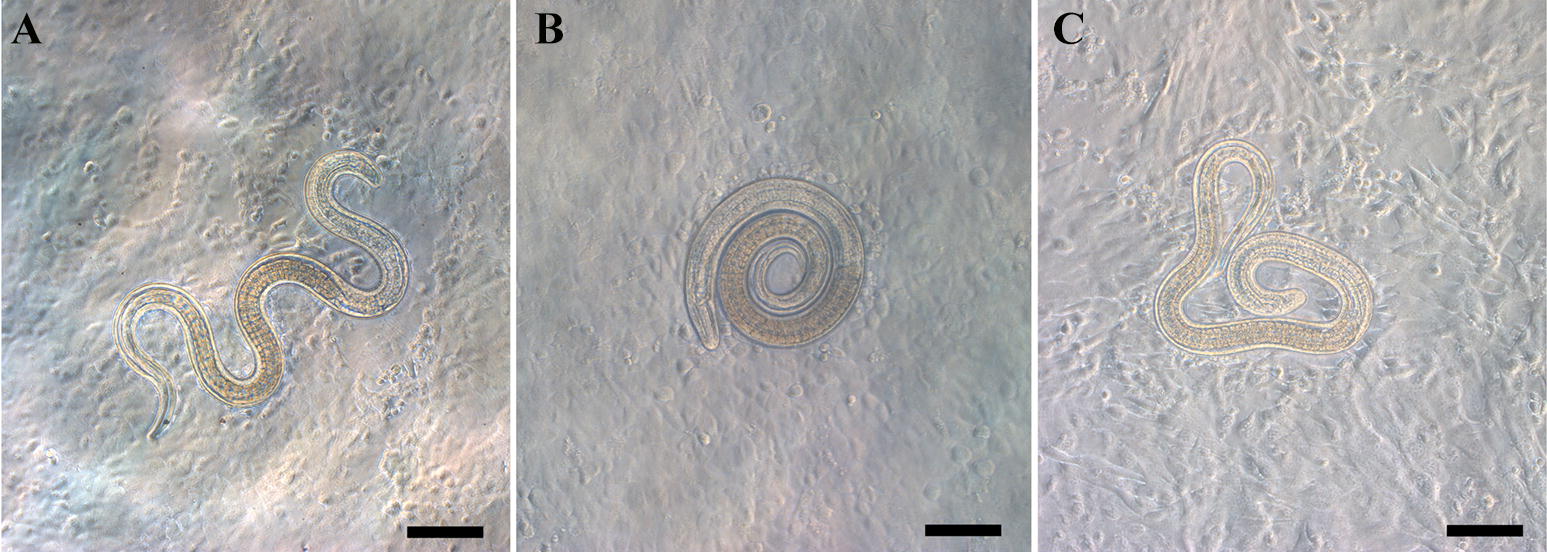
**The process of IIL invasion into the intestinal epithelium in vitro. A***T. spiralis* IIL were shown to invade the epithelial cell monolayer. Larvae penetrated IEC cells and migrated through them, leaving behind trails of dead cells and damaging the IEC monolayer. **B** Non-invaded larvae on the IEC monolayer. Nematodes that stayed coiled on the surface of the IEC monolayer or suspended in the medium were counted as non-invaded larvae. The IEC monolayer was intact. **C** Non-invaded larvae on the C2C12 cell monolayer. The larvae on the C2C12 monolayer were coiled. The C2C12 cell monolayer was not damaged. Scale bars: 100 μm.

One hundred IIL of each treatment group were cultured in different semi-solid media in 5% CO_2_ at 37 °C for 2 h. Larval invasion was observed under a microscope (Olympus, Japan). Larvae that invaded or migrated in the IEC monolayer, forming trails, leaving dead cells behind and destroying the IEC monolayer, were counted as invaded larvae (Figure 7A). Larvae suspended in the medium or remaining coiled on the cell monolayer surface, not damaging the cells, were defined as uninvaded larvae (Figure 7B). C2C12 cells were not susceptible to larval invasion [38]; non-invaded larva were observed on the C2C12 monolayer (Figure 7C) as the control for larval invasion of the IEC monolayer.

**Figure 8:**
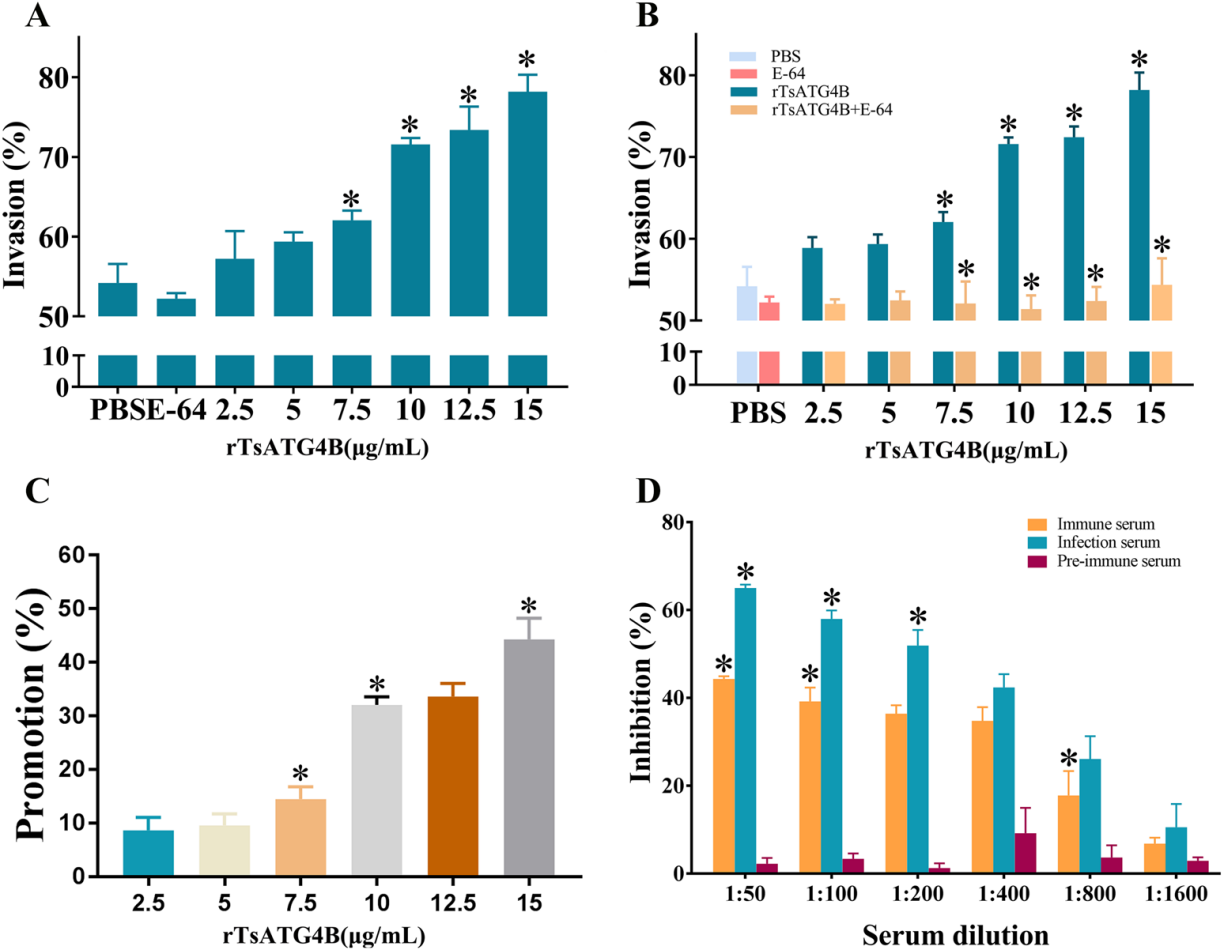
**Promotive effect of rTsATG4B protein and inhibitory effect of anti-rTsATG4B serum on larval invasion of the IEC monolayer in vitro. A** Promotive effect of rTsATG4B on larval invasion. **B** The promotive effect on larval invasion was diminished when rTsATG4B was pre-incubated with E-64 (10 μM, 1:1); **c** The promotion rate of rTsATG4B protein on *T. spiralis* larval invasion of the IEC monolayer in vitro. Data were compared with the PBS group as the control. **D** The inhibition rate of anti-rTsATG4B serum showed a decreasing trend with increasing dilution and displayed a dose-dependent relationship with anti-rTsATG4B serum Data were compared with the PBS group as the control. *P < 0.01.

The results revealed that increasing amounts of rTsATG4B significantly accelerated the invasion of worms into the monolayer (Figure 8A). The acceleration effect had a dose-dependent relationship with rTsATG4B (r = 0.97, *P *< 0.01), and the amount of invasion displayed an increasing trend as the rTsATG4B dose increased (F = 52.623, *P* < 0.01) (Figure 8B). On the other hand, the acceleration effect was diminished when rTsATG4B was pre-incubated with the same dose of E-64 at 37 °C for 30 min before replenishment of the medium (Figure 8C). In contrast, anti-rTsATG4B serum and infection serum inhibited larval invasion of IECs (χ^2^ = 112.418, *P* < 0.01) compared with that in the normal serum group. The inhibitory effect of anti-rTsATG4B serum showed a decreasing trend along with an increase in the dilution; thus, invasion also demonstrated a dose-dependent relationship with anti-rTsATG4B serum (r = 0.96, *P* < 0.01) (Figure 8D). The data are expressed as the standardized suppression rate (%) relative to the PBS group.

## Discussion

Research has revealed that cysteine proteases are vital contributors to the invasion and migration of host tissues by parasitic organisms, which has attracted scientists’ attention. Cysteine proteases degrade host tissues and promote penetration and migration throughout various parasite developmental stages. They also have critical roles in pathogenesis, as they are involved in nutrition, moulting, metabolism and immune modulation [12, 34]. In *Trichuris*, Drake et al. [35] first used a fluorogenic peptidyl substrate to investigate the activity of a cysteine protease in soluble extracts of *Trichuris muris* (*T. muris*) AW in nutrition and invasion. Hasnain et al. [39] revealed that proteolytic activity in *T. muris* ESPs was necessary for degrading host intestinal tissues. However, this group also indicated that cysteine proteases exist in ESPs and disrupt the network of polymeric mucin. Based on these preliminary studies, this project was instigated to study the role played by TsATG4B in larval invasion of the intestine of the host, and we found promising results in invasion assays.

Cysteine peptidases hydrolyse peptide bonds in a polypeptide chain via a mechanism in which the sulfhydryl group of a cysteine residue at the active centre acts as a nucleophile. Cysteine peptidases can be divided into 14 different clans, and each clan has a tertiary fold unique to that clan. Cysteine peptidases are generally active in acidic environments, such as animal lysosomes or plant vacuoles, which limits their applications. A cysteine peptidase may be an endopeptidase, an aminopeptidase, a carboxypeptidase, a dipeptidyl peptidase or an ω-peptidase. Their bioactivities are inhibited by thiol chelating agents such as iodoacetate, iodoacetic acid, and N-ethylmaleimide [40, 41]. We investigated whether TsATG4B is recognized by early *T. spiralis* infection sera and ascertained the biochemical characteristics and functions of TsATG4B during invasion of the host intestine [26]. Only the first 17 amino acid residues, which were predicted to be a non-signal sequence, were deleted, and the rTsATG4B protein was expressed in *Escherichia coli*. We speculated that a signal peptide exists in TsATG4B, although it was not predicted by SignalP4.1 Server. The molecular weight of the rTsATG4B protein was approximately 43 kDa, consistent with the calculated size.

TsATG4B belongs to the C54 peptidase family (Aut2 peptidase family, clan CA). Clan CA includes proteins with papain-like folds. There is a catalytic triad that occurs in the following order: Cys/His/Asn (or Asp). The fourth residue, usually Gln, is important for stabilizing the acyl intermediate formation during the catalytic process and is upstream of the Cys active site. Folding forms 2 subdomains separated by active sites. Peptidases in clan CA are generally sensitive to small molecule E-64 inhibitors, while E-64 inhibitors are ineffective against other cysteine peptidase families [25, 42]. In our studies, we successfully refolded rTsATG4B. The 1-D zymography results showed that rTsATG4B exhibits gelatinolytic activity at pH 5.2 and that this activity was eliminated by the cysteine protease inhibitor E-64. This gelatinolytic activity may be related to digestion of the host tissue during invasion, migration and pathogenesis [43–47]. More interestingly, another *T. spiralis* cysteine peptidase was investigated as a putative cathepsin F-like protease (TsCF1) by Qu et al. [23]. In their studies, they demonstrated with a Z-Phe-Arg-AMC substrate that rTsCF1 has enzymatic activity after renaturation and that this activity was inhibited by the cysteine protease inhibitor E-64. This study provides valuable references for us to further study the enzymatic activity of TsATG4B. This result prompted us to believe that using fluorescent peptides as substrates to study the activity of proteases is worth investigating and that it is crucial to determine the optimal pH, kinetic parameters and so on.

The qPCR analysis results showed that the level of transcription in 6 days AW was significantly greater than that in the other stages. The Western blot results showed that the rTsATG4B protein was recognized by anti-rTsATG4B serum and infection serum for 20–30 days. Somatic proteins of NBL, ML, IIL, 3 days AW, and 6 days AW and ES proteins of ML were probed with anti-TsATG4B serum for the TsATG4B protein. The IFT results also indicated the expression of TsATG4B at different stages. The results showed that immunostaining was detected on the surfaces, cuticles, and stichosomes and in worm embryos and revealed that TsATG4B was expressed at the levels of transcription and translation throughout all developmental stages of *T. spiralis*. In our work, native TsATG4B was detected in somatic proteins and ES proteins of ML by Western blotting, indicating that TsATG4B is a somatic protein component of the parasite and likely to be a secreted protein. Additionally, previous studies showed that nudix hydrolase, glutathione S-transferase, serine proteases and aminopeptidase are expressed in various stages of *T. spiralis* development [31, 48, 49]. In other parasites, notably *Fasciola hepatica* and *Schistosoma* [50, 51], the expression of various types of cysteine proteases in different developmental stages has been detected, suggesting that these cysteine proteases may have specific functions in each stage, such as the disrupting different tissue barriers or contrasting protein composition. Therefore, we gained insight into the interaction between proteases and barrier cells. In fact, our observations via IFA and confocal microscopy demonstrated the binding and cellular localization of TsATG4B in IECs occurred at the membrane and in the cytoplasm of IECs. The Far-Western blot results showed that approximately 10 bands from IEC lysates pre-incubated with rTsATG4B were recognized by anti-rTsATG4B serum and infectious serum but not by normal serum. This result further confirmed the interactions between TsATG4B and barrier cells. Only these interactions illustrated the important role of TsATG4B in the processes of *T. spiralis* invasion, colonization, parasite escape, and so on.

The in vitro invasion assay results indicated that the inhibitory effect of anti-rTsATG4B serum was reduced with reducing serum concentration (increasing dilution). Infectious serum and anti-rTsATG4B serum significantly inhibited larval invasion of IECs. Studies have shown that some immune sera against recombinant *Trichinella* proteins effectively protect the intestinal epithelium against larval invasion [49, 52, 53]. The inhibition mechanism of the antibody against recombinant *Trichinella* protein could be related to the formation of immune complexes at the end of the larval cephalic stage, which was able to prevent larval invasion [54]. When rTsATG4B was added to the medium, there was an obvious promotion of worm invasion of the IEC monolayer. This promotive effect displayed an increasing trend and a dose-dependent relationship with rTsATG4B. Moreover, the promotive effect of rTsATG4B on larval invasion was diminished when rTsATG4B was pre-incubated with E-64. The excystation of trophozoites of *Giardia lamblia* and metacercariae of *Paragonimus westermani* is inhibited or blocked by E-64 in a dose-dependent manner, suggesting that cysteine proteases of these parasites are involved in excystment [55]. Our results also demonstrated that rTsATG4B plays an important role in larval invasion, which may be due to its hydrolytic activity on the host’s intestinal epithelium. Since this hydrolysis process can be suppressed by E-64, the TsATG4B protein appears to be an important therapeutic target for trichinellosis.

Our results showed that the transcription and expression of TsATG4B was maintained through all stages of *T. spiralis* development. TsATG4B was mainly localized on the surfaces, cuticles and stichosomes of the nematode. Refolded rTsATG4B displayed enzymatic activity, which was eliminated by E-64. These findings showed the interaction with enterocytes of the host and verified the promotive effect on larval invasion of IECs, which was inhibited by E-64. The anti-rTsATG4B antibody inhibited larval invasion of the IEC monolayer in a dose-dependent manner, which suggested that TsATG4B plays an important role in larval invasion of the intestinal epithelium.

## Supplementary information

**Additional file 1.Analysis of the amplicons of the TsATG4B gene and T. spiralis 18S rRNA gene by 5% agarose gel electrophoresis**. **A** The amplicon of the *TsATG4B* gene by agarose gel electrophoresis (76 bp). M: DL 500 DNA marker; 1: ML; 2: IIL; 3: 3 d AW; 4: 6 d AW; 5: NBL; **B** The amplicon of the T. spiralis 18S rRNA gene by agarose gel electrophoresis (117 bp). M: DL 500 DNA marker; 1: ML; 2: IIL; 3: 3 d AW; 4: 6 d AW; 5: NBL.

**Additional file 2.Negative control for the TsATG4B immunolocalization assay**. The immunolocalization of TsATG4B in intact ML (**A**, **B**). Paraffin sections (**C**, **D**) incubated with normal mouse serum (**A**, **C**) and PBS (**B**, **D**) served as the negative controls. Scale bars: 50 μm.

**Additional file 3.Analysis of IEC lysates and C2C12 lysates by SDS-PAGE**. Lane M: protein molecular weight marker; lane 1: IEC lysates; lane 2: C2C12 lysates.

## Data Availability

This work was mainly supported by the Key Scientific Research Project of Colleges and Universities in Henan Province (2014-J-129-R05/08).

## Abbreviations

AW: adult worms
3 d AW: 3-day-old adult worms
6 d AW: 6-day-old adult worms
DAB: 3, 3′-diaminobenzidine tetrahydrochloride
EDTA: ethylenediaminetetraacetic acid
ES: excretory-secretory
HRP: horseradish peroxidase
IEC: intestinal epithelial cell
IFA: immunofluorescence assay
IFT: immunofluorescence test
IIL: intestinal infective larvae
IPTG: isopropyl β-d-1-thiogalactopyranoside
ML: muscle larvae
NBL: newborn larvae
ORF: open reading frame
PI: propidium iodide
*T. muris*: *Trichuris muris*
TsATG4B: *Trichinella spiralis* cysteine protease ATG4B
*T. spiralis*: *Trichinella spiralis*
rTsATG4B: rsecombinant *Trichinella spiralis* cysteine protease ATG4B
