# Supplementary figures and images for: The cysteine protease ATG4B of Trichinella spiralis promotes larval invasion into the intestine of the host
Source: Vet Res. 2020 May 24;51:69. doi: 10.1186/s13567-020-00791-z (PMC7245929; doi:10.1186/s13567-020-00791-z)

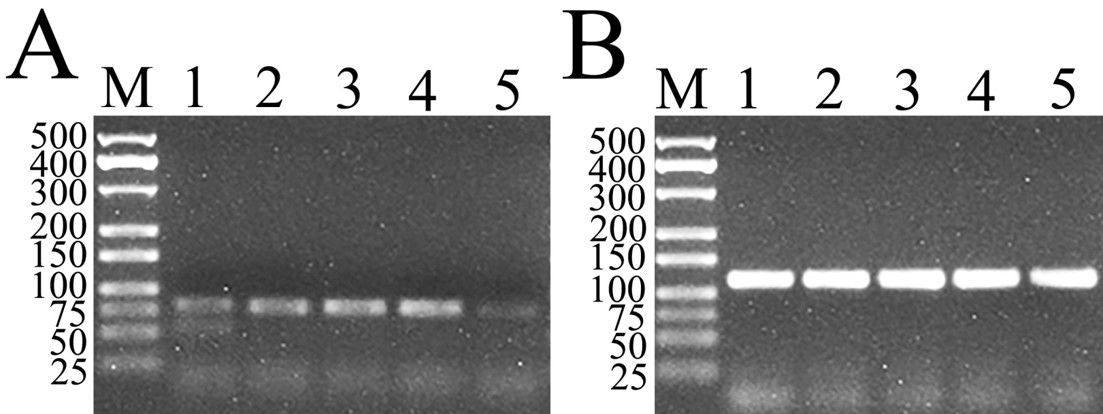

Supplement: Supplementary file 1 — Additional file 1.Analysis of the amplicons of the TsATG4B gene and T. spiralis 18S rRNA gene by 5% agarose gel electrophoresis. A The amplicon of the TsATG4B gene by agarose gel electrophoresis (76 bp). M: DL 500 DNA marker; 1: ML; 2: IIL; 3: 3 d AW; 4: 6 d AW; 5: NBL; B The amplicon of the T. spiralis 18S rRNA gene by agarose gel electrophoresis (117 bp). M: DL 500 DNA marker; 1: ML; 2: IIL; 3: 3 d AW; 4: 6 d AW; 5: NBL. [file 13567_2020_791_MOESM1_ESM.jpg]

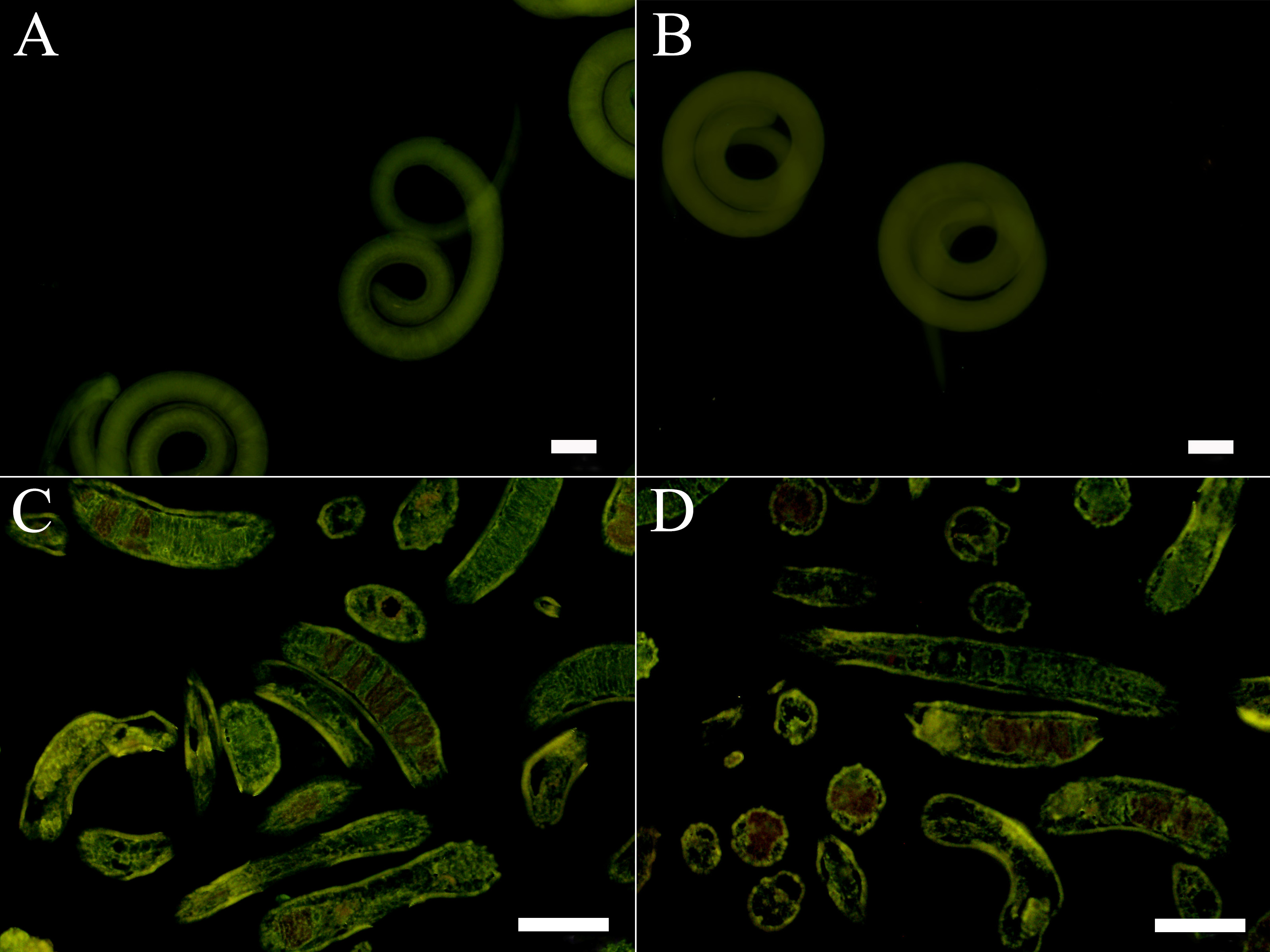

Supplement: Supplementary file 2 — Additional file 2.Negative control for the TsATG4B immunolocalization assay. The immunolocalization of TsATG4B in intact ML (A, B). Paraffin sections (C, D) incubated with normal mouse serum (A, C) and PBS (B, D) served as the negative controls. Scale bars: 50 μm. [file 13567_2020_791_MOESM2_ESM.jpg]

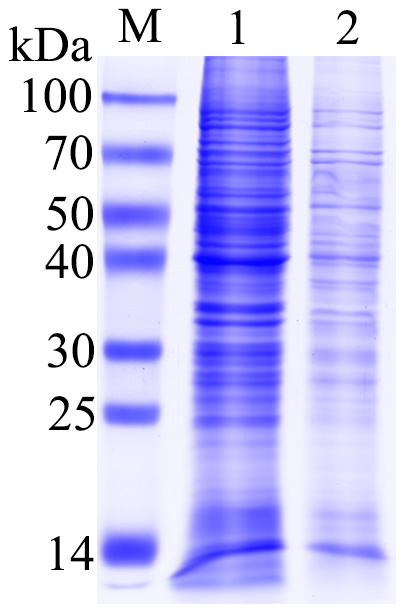

Supplement: Supplementary file 3 — Additional file 3.Analysis of IEC lysates and C2C12 lysates by SDS-PAGE. Lane M: protein molecular weight marker; lane 1: IEC lysates; lane 2: C2C12 lysates. [file 13567_2020_791_MOESM3_ESM.jpg]
